# Beyond First Impressions

**DOI:** 10.3201/eid2108.AC2108

**Published:** 2015-08

**Authors:** Byron Breedlove, Jared Friedberg

**Affiliations:** Centers for Disease Control and Prevention, Atlanta, Georgia, USA

**Keywords:** art science connection, emerging infectious diseases, disease surveillance, art and medicine, Cornelius Norbertus Gijsbrechts, Trompe l’oeil with Studio Wall and Vanitas Still Life, Statens Museum for Kunst, Copenhagen, trompe l’oeil, Beyond First Impressions, about the cover

**Figure:**
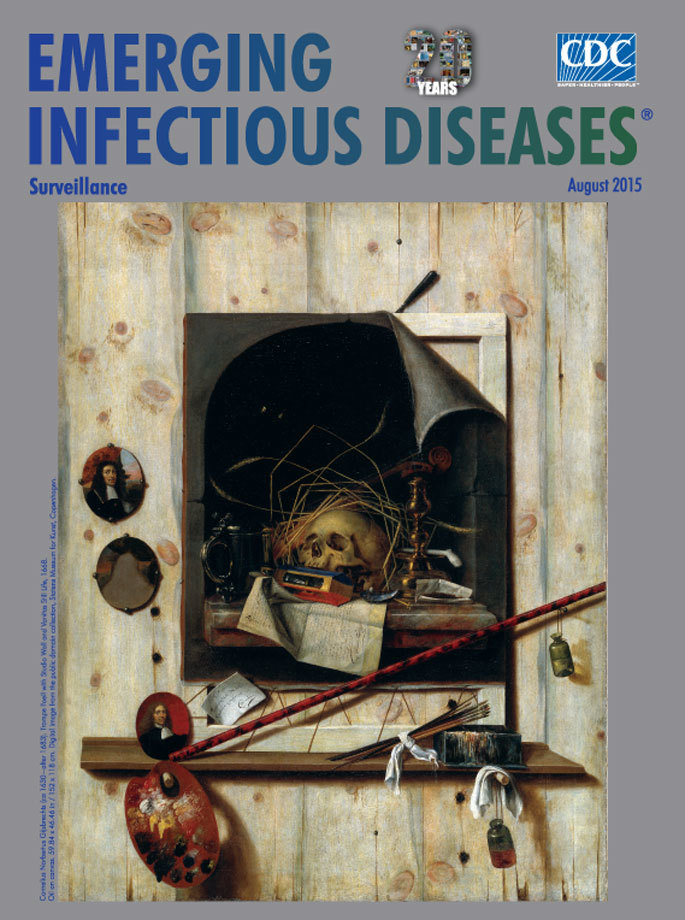
**Cornelius Norbertus Gijsbrechts (ca 1630−after 1683) 1668. Trompe l’oeil with Studio Wall and Vanitas Still Life, oil on canvas. 59.84 × 46.46 in/152 × 118 cm.** Digital image from the public domain collection, Statens Museum for Kunst, Copenhagen

Few details endure about the life and family circumstances of Flemish painter Cornelius Norbertus Gijsbrechts. We know that he was born in Antwerp, Belgium, and his talent earned him a position of court painter with two Danish kings, Frederick III (1609−1675) and Christian V (1646−1699). In 1660, Gijsbrechts was accorded membership in the prominent, influential Guild of St. Luke. Over his career, the artist worked in various locations, including Antwerp, Regensburg, Hamburg, and Copenhagen, and it was in the latter city where he created his signature series of trompe l’oeil (deception of the eye) paintings during 1668−1672.

Trompe l’oeil, a subgenre of still life painting, embraces use of realistic imagery to create an optical illusion of depth in works that can be perceived as real, three-dimensional objects rather than flat paintings. It flourished from the Renaissance onward, though murals of trompe l'oeil art are found among the ruins of Pompeii and Herculaneum and remain popular today. According to the National Gallery of Art, “The discovery of perspective in fifteenth-century Italy and advancements in the science of optics in the seventeenth-century Netherlands enabled artists to render objects and spaces with eye-fooling exactitude.”

This month’s cover painting *Trompe l’oeil with Studio Wall and Vanitas Still Life* is a prime example of this inventive subterfuge and also of a vanitas painting, a meditation on mortality. Gijsbrechts makes a skull the focal point of the painting, ensuring no one misses his allusion to the temporal nature of life. Other symbols supports the theme of transience: smoke trailing from an extinguished candle and an hourglass tipped on its side symbolize death; a violin, tankard, clay pipe, and tobacco represent fleeting pleasures. The unity of these symbols represent the ebb and flow of birth, death, and resurrection, encouraging both a wistful examination of life’s purpose and a solemn acceptance of death’s finality.

Gijsbrechts pulls the scene back and reveals the vanitas on a temporary canvas hung on a wooden wall, surrounded by the tools of his craft, including brushes and a palette featuring the colors he used in his construction of the painting. A miniature self-portrait affixed to the frame reminds viewers that art endures beyond the life of the artist. Those who fall for the deception and perceive the vanitas as the sole painting miss the message that Gijsbrechts intends: nothing is what it seems without context.

In trompe l’oeil, the background imagery surrounding the painting’s center provides perspective and clues that engage viewers and contribute to understanding the overall work. Disease surveillance for emerging infections relies on a somewhat similar, albeit more complicated process. To gain perspective, one must assess a complex but incomplete set of intertwined, dynamic factors, which may include antimicrobial drug resistance, climate change, food production practices, global mobility, the route(s) of transmission of the etiologic agents, and ecology of the pathogens.

By their nature, surveillance data are representative of disease in populations, and they are always incomplete and sometimes inaccurate. It takes a critical, well-prepared mind to properly interpret and analyze the meaning and significance of surveillance data or unravel clues about how an emerging infectious disease spreads. Gijsbrechts’ portrait displays the tools and medium that he used to construct his illusion, but not everyone who views the painting will come to this realization. The experience of viewing a trompe l’oeil reminds us that seeing beyond our initial assumptions—whether we are studying art, investigating the outbreak of an emerging infection, or analyzing reams of data—requires that we connect background information, delve beyond the surface, and recognize patterns and aberrations.
